# Image Target Recognition via Mixed Feature-Based Joint Sparse Representation

**DOI:** 10.1155/2020/8887453

**Published:** 2020-08-10

**Authors:** Xin Wang, Can Tang, Ji Li, Peng Zhang, Wei Wang

**Affiliations:** ^1^School of Computer and Communication Engineering, Changsha University of Science and Technology, Changsha 410114, China; ^2^School of Electronics and Communications Engineering, Sun Yat-sen University, Shenzhen 518107, China

## Abstract

An image target recognition approach based on mixed features and adaptive weighted joint sparse representation is proposed in this paper. This method is robust to the illumination variation, deformation, and rotation of the target image. It is a data-lightweight classification framework, which can recognize targets well with few training samples. First, Gabor wavelet transform and convolutional neural network (CNN) are used to extract the Gabor wavelet features and deep features of training samples and test samples, respectively. Then, the contribution weights of the Gabor wavelet feature vector and the deep feature vector are calculated. After adaptive weighted reconstruction, we can form the mixed features and obtain the training sample feature set and test sample feature set. Aiming at the high-dimensional problem of mixed features, we use principal component analysis (PCA) to reduce the dimensions. Lastly, the public features and private features of images are extracted from the training sample feature set so as to construct the joint feature dictionary. Based on joint feature dictionary, the sparse representation based classifier (SRC) is used to recognize the targets. The experiments on different datasets show that this approach is superior to some other advanced methods.

## 1. Introduction

In recent years, sparse representation classification (SRC) approach has successfully been used in the field of image recognition. Compared with other methods, SRC is robust to illumination, occlusion, and noise. In the feature extraction stage, the traditional image recognition methods based on sparse representation usually use the original samples directly or the low-dimensional samples after dimensionality reduction as the atoms to construct the dictionary. However, the dictionary constructed in this way cannot effectively represent the test samples, and it is difficult to make full use of the information hidden between the training samples. So, many scholars began to study the use of various features in the construction of dictionaries.

Gabor transform is a windowed Fourier transform, first proposed by Lee [1]. Later, Gabor wavelet transform was put forward by combining Gabor transform with wavelet transform. Different from the traditional Fourier transform, Gabor wavelet transform can easily adjust the frequency and direction of the filter, so the signal features obtained by Gabor wavelet transform have good discrimination in the time-space domain and the frequency domain. Using Gabor wavelet transform to extract the features of the original samples for sparse representation classification can avoid the problems caused by the direct construction of dictionaries from the original samples to some extent. Lu and Zhang proposed a face recognition method based on discriminant dictionary learning, which obtained the Gabor amplitude images of the faces through Gabor filter. Then, they used the Gabor amplitude images to construct a new dictionary for sparse representation classification, which improved the recognition rate of the face images in the uncontrolled environment [2].

As a popular image classification and recognition framework, convolutional neural network (CNN) has attracted a great deal of scholarly attention. However, CNN needs a large number of samples for training. In reality, many samples are not easily obtained, and the cost of CNN parameters adjustment is also large. CNN can extract a variety of features, such as texture, shape, color, and topology at the same time, so it is also very suitable to be used as a tool to extract image features [3, 4]. Zhang et al. proposed a CNN-GRNN model for image classification and recognition [5]. The model used CNN to extract image features and then used general regression neural network (GRNN) for classification and recognition. The deep features extracted by CNN enabled the method to have a good recognition effect. In order to better extract the features, the image superresolution can be applied for the image reconstruction first [6].

When Gabor wavelet transform is used to extract features for target recognition, the impact of light condition transformation on recognition can be reduced. At the same time, it has better robustness for image deformation and rotation to some extent. Therefore, this paper proposes an image target recognition method based on mixed features and joint sparse representation (M-JSR). The Gabor wavelet feature extracted by Gabor wavelet transform and the deep feature extracted by CNN were combined to form the hybrid feature and carry out adaptive weighting and PCA dimensionally reduction for mixed features and finally combined with the joint sparse model for classification recognition. The problem of poor representation ability of the original dictionary is avoided by building the dictionary with mixed features instead of the original sample. Compared with using CNN for classification recognition, M-JSR does not require a large number of training samples nor does it need a lot of time to adjust parameters. Moreover, the joint sparsity model divides the dictionary into the public features part and the private features part, so that the dictionary has better discrimination ability, and thus improves the recognition accuracy.

## 2. Feature Extraction

### 2.1. Gabor Wavelet Feature Extraction

Gabor wavelet transform has unique advantages in the representation, and analysis of image signals for images can be processed in different scales and directions. In simple terms, Gabor wavelet transform is used to convolve a set of Gabor filter functions with a given image signal.

In general, the two-dimensional Gabor function can be expressed as [1]

(1) $$\begin{array}{c} \psi_{u,v}\left(m,n\right)=\frac{k^{2}}{\sigma^{2}}\exp\left(-\frac{k^{2}{\left(m+n\right)}^{2}}{2\sigma^{2}}\right)\cdot \left[\exp\left(ik\cdot \left(\frac{m}{n}\right)\right)-\exp\left(-\frac{\sigma^{2}}{2}\right)\right], \end{array}$$

where *k*=*k*_*v*_(cos*θ*, sin*θ*)^*T*^,*θ*=*πu*/8 represents the direction of the filter, *k*_*v*_=*k*_max_/*f*^*v*^, *k*_max_represents the maximum frequency, *f* is the interval factor of the kernel function in the frequency domain, and *u* and *v* represent the direction and scale of Gabor wavelet, respectively. Researches show that using 5 scales (*v*=0,1,2,3,  *and* 4) and 8 directions (*u*=0,1,2,3,4,5,6,  *and* 7) can get the best effect [7]. *m* and *n* represent the spatial coordinates of the image, *σ*is the radius of the Gaussian function (which is the size of the two-dimensional Gabor wavelet) and *i* is a complex number operator.

Assume the input image is *I*=(*m*, *n*), then

(2) $$\begin{array}{c} F_{u,v}\left(m,n\right)=I\left(m,n\right)⊗\psi_{u,v}\left(m,n\right), \end{array}$$

where *F*_*u*,*v*_(*m*, *n*) represents the Gabor wavelet features of the image *I*=(*m*, *n*).

### 2.2. Deep Feature Extraction

Convolutional neural network (CNN) [8] is a feedforward neural network, which is essentially a multilayer perceptron. A complete convolutional neural network consists of the input layer, the convolutional layer, the subsampling layer (pooling layer), and the fully connected layer. The convolution layer is used to extract the features of the input data, and it generally contains multiple convolution kernels. The pooling layer mainly compresses the features which are extracted by the convolution layer to decrease the complexity of network computing and improve the robustness. The full connection layer combines the previously extracted features nonlinearly and sends the output value to the classifier, such as softmax classifier. Therefore, in addition to image classification, CNN can also be used as a tool to extract image features.

**Figure 1:**
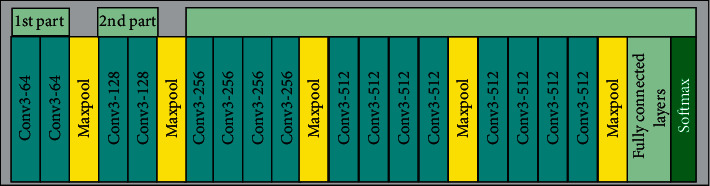
Structure of VGG19.

For extracting sparse features, we draw on the viewpoint of the literature [9–11] about network design. Visual geometry group networks (VGGNets) proposed by Simonyan and Zisserman have significantly improved image recognition performance by deepening the network to 19 layers. VGG19 network is used to extract deep features, and its structure is shown in Figure 1. In VGG19, the convolution filters are set to 3×3, and the max pooling is 2×2 with stride 2. VGG19 has better performance than other convolutional network models in extracting target features. As shown in Figure 1, the number of convolution kernels at the next layer is doubled when the size of the feature map is reduced by half through the max pooling layer. VGG19 ends with three fully connected layers and softmax function.

**Figure 2:**
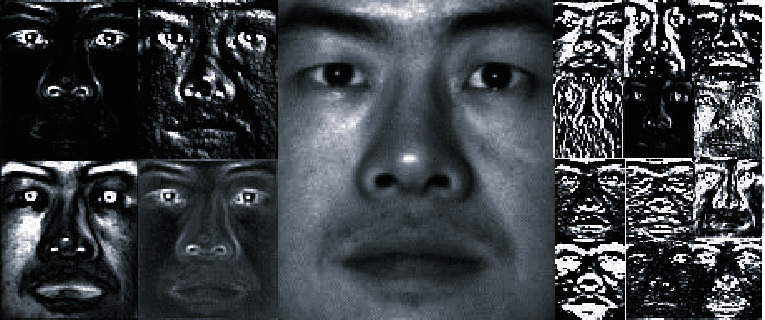
Samples of deep features.

The convolution kernel of CNN convolutional layer can automatically extract complex global and local features from the image. The convolution kernels of shallow layers in the CNN network extract mostly texture and detail features. Relatively speaking, the deeper the layers are, the more representative the extracted features will be, while the resolution of the feature maps will become lower. As shown in Figure 2, the middle part is the original figure, the left side is the feature extracted by the convolution layer of the first part of VGG19 network, and the right side is the feature extracted by the convolution layer of the second part of VGG19 network.

## 3. Joint Sparsity Model

### 3.1. Joint Sparsity Model

The joint sparsity model (JSM) was originally used for the coding of multiple related signals in distributed compressed sensing scenes [12]. In JSM, according to the intrasignal and the intersignal correlation, a group of related signals can be regarded as a signal set. Then, each signal in the signal set can be jointly represented by the public feature of this type of signal and its own private feature, such as formula (3). Both public and private features can be sparsely represented on the same sparse basis.

(3) $$\begin{array}{c} y_{j}=z_{c}+z_{j},\;j\in \left\{1,2,3,\ldots ,J\right\}, \end{array}$$

where*y*_*j*_is the *j*th signal in a certain type of signal, *z*_*c*_represents the public feature of this type of signal, and *z*_*j*_represents the private feature of the *j*th signal.

If all the samples can be classified into *K* categories, and each containing *J* samples, the *j*th sample of class *i* can be represented as*y*_*i*,*j*_. After putting all the samples of class *i* into one set, we can represent it as*y*_*i*_=[*y*_*i*,1_, *y*_*i*,2_,…,*y*_*i*,*J*_]^*T*^. Then, as shown in formula (4), the *j*th sample of class *i* can be represented by a combination of public and private features, thus greatly reducing the required storage space:

(4) $$\begin{array}{c} y_{i,j}=z_{i}^{c}+z_{i,j}^{i}, \end{array}$$

where *z*_*i*_^*c*^is the public feature of all samples in class *i* and*z*_*i*,*j*_^*i*^is the private feature of the *j*th sample of class *i* [13]. Assuming that the samples can be sparsely represented on the orthogonal basisΨ ∈ *R*^*N*×*N*^, formula (4) can be expressed as

(5) $$\begin{array}{c} \theta_{i,j}=\Psi y_{i,j}=\Psi z_{i}^{c}+\Psi z_{i,j}^{i}=\theta_{i}^{c}+\theta_{i,j}^{i}, \end{array}$$

where *θ*_*i*_^*c*^=Ψ*z*_*i*_^*c*^represents the sparse representation of the public part onΨ and *θ*_*i*,*j*_^*i*^=Ψ*z*_*i*,*j*_^*i*^represents the sparse representation of the private part onΨ. Through left multiplyingΨ^*T*^, Ψ^*T*^*θ*_*i*,*j*_=Ψ^*T*^*θ*_*i*_^*c*^+Ψ^*T*^*θ*_*i*,*j*_^*i*^=*z*_*i*_^*c*^+*z*_*i*,*j*_^*i*^=*y*_*i*,*j*_, the images of class *i* can be represented as

(6) $$\begin{array}{c} \left[\begin{array}{c} y_{i,1}\\ y_{i,2}\\ \vdots \\ \vdots \\ y_{i,J} \end{array}\right]=\left[\begin{array}{ccccc} \Psi^{T} & \Psi^{T} & 0 & \cdots & 0\\ \Psi^{T} & 0 & \Psi^{T} & 0 & \vdots \\ \vdots & \vdots & 0 & \ddots & 0\\ \Psi^{T} & 0 & \cdots & 0 & \Psi^{T} \end{array}\right]\cdot \left[\begin{array}{c} \theta_{i}^{c}\\ \theta_{i,1}^{i}\\ \theta_{i,2}^{i}\\ \vdots \\ \vdots \\ \theta_{i,J}^{i} \end{array}\right]. \end{array}$$

After simplifying, formula (6) can be expressed as

(7) $$\begin{array}{c} y_{i}=\tilde{\Psi }W_{i}, \end{array}$$

where *y*_*i*_=[*y*_*i*,1_, *y*_*i*,2_,…,*y*_*i*,*j*_]^*T*^, $W_{i}={\left[\begin{array}{ccccc} \theta_{i}^{c} & \theta_{i,1}^{i} & \theta_{i,2}^{i} & \ldots & \theta_{i,j}^{i} \end{array}\right]}^{T}$, and $\tilde{\Psi }=\left[A,B\right]$ represents an overcomplete dictionary that contains two parts:$A=\left[\begin{array}{cccc} \Psi^{T} & \Psi^{T} & \cdots & \Psi^{T} \end{array}\right]$ and *B*=diag(*A*).*W*_*i*_can be obtained by solving the *l*_1_ minimization problem as follows:

(8) $$\begin{array}{c} W_{i}=\arg\min{\left\|W_{i}\right\|}_{1},\\ \text{s}.\text{t}.\;y_{i}=\tilde{\Psi }W_{i}. \end{array}$$

After obtaining*W*_*i*_, according to the inverse transformation, the public features of all images of class *i* and the private features of each image in the Ψ domain can be obtained as

(9) $$\begin{array}{c} \begin{array}{c} z_{i}^{c}=\Psi^{T}\theta_{i}^{c},\\ z_{i,j}^{i}=\Psi^{T}\theta_{i,j}^{i}. \end{array} \end{array}$$

Combining all public and private features can get the joint feature dictionary *D*:

(10) $$\begin{array}{c} D=\left[z_{1}^{c},z_{2}^{c},\ldots ,z_{K}^{c},z_{1,1}^{1},\ldots ,z_{1,J}^{1},z_{2,1}^{2},\ldots ,z_{2,J}^{2},\ldots ,\ldots ,z_{K,1}^{K},\ldots z_{K,J}^{K}\right]. \end{array}$$

Finally, according to the sparse representation classification method, the target can be classified by the following formula:

(11) $$\begin{array}{c} \text{class}\left(i\right)=\arg\underset{i}{\min}{\left\|y-D\delta_{i}\left(x^{\prime }\right)\right\|}_{2}, \end{array}$$

where *x*′represents the sparse coefficient vector that can be reconstructed from *y* with the dictionary.

### 3.2. Adaptive Weighted Reconstruction

When using SRC, the information carried by atoms in different dictionaries is mainly used to sparse reconstruction. Therefore, in order to improve the recognition accuracy, the atoms with more target information can be screened out by calculating the variance or standard deviation. And, the contribution ability of these atoms can be artificially improved to make the dictionary more discriminant [14].

Suppose *F*=[*F*_1_, *F*_2_,…,*F*_*n*_]^*T*^ is a vector which extracted from an image, and then it can be modified by the following formula:

(12) $$\begin{array}{c} F^{\prime }=\left[F_{1}^{\prime }=\frac{\left|F_{1}-\bar{F}\right|}{\bar{F}}F_{1},F_{2}^{\prime }=\frac{\left|F_{2}-\bar{F}\right|}{\bar{F}}F_{2},\cdots ,F_{n}^{\prime }=\frac{\left|F_{n}-\bar{F}\right|}{\bar{F}}F_{n}\right], \end{array}$$

where $\bar{F}=\left(F_{1}+F_{2}+\cdots +F_{n}\right)/n$, *F*_*i*_′represents the *ith* feature after weighted reconstruction. After the above processing, the variance between the feature vectors will increase to a certain extent. The feature dictionary contains more recognition information, which can improve the discrimination ability of the dictionary.

## 4. Framework of Mixed Feature-Based Joint Sparse Representation (M-JCR)

**Figure 3:**
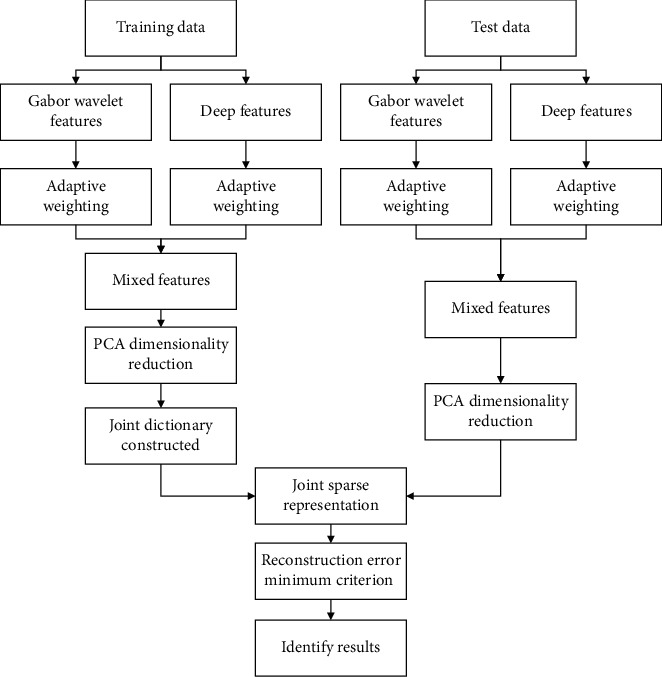
The algorithm framework of M-JSR.

The algorithm framework is shown in Figure 3. First, Gabor wavelet features and deep features are combined into mixed features. Then, the joint sparsity model is used to extract public feature and private feature to build joint dictionary, and the test samples are sparse reconstructed. Finally, the target can be identified on the basis of the minimum reconstruction error criterion.

The specific steps of M-JSR are as follows:

1. Gabor wavelet transform is used to extract Gabor wavelet features of training images and test images, and CNN is used to extract deep features of training images and test images.
2. The Gabor wavelet feature and deep feature are adaptively weighted to form the mixed feature set, and the mixed feature is dimensionally reduced by PCA.
3. The public feature of each class and the private feature of each image are extracted from the training image feature set. The public features are formed into a matrix *M*, and all private features are arranged into a matrix *N* to form a joint feature dictionary*D*=[*M*, *N*], as shown in Formula (10).
4. The mixed feature vector of the test image is sparsely represented on the joint feature dictionary to get the sparse coefficient*x*′, and the mixed feature vector of the test image is reconstructed.
5. Finally, the recognition result is obtained through Formula (11).

## 5. Experiments and Analysis

In this paper, M-JSR is verified on face images, AR data set, and remote sensing images, respectively. The platform used in the experiment is Matlab R2017a. The computer is configured as Intel Core i5-3210M@2.5 GHz, and the memory is 4 GB. The experimental results are the average values of 10 experiments.

### 5.1. Face Image Recognition

In this part, two face datasets of AR [15] and Extended YaleB [16] are selected, and our experiment results are compared with SRC [17], extended SRC (ESRC) [18], low-rank matrix recovery method (LR) [19], discriminative low-rank representation method (DLRR) [20], sparse dictionary decomposition method (SDD) [21], adaptive weighting joint sparse representation method (AJSR) [14], and deep feature-based adaptive joint sparse representation(D-AJSR) [22], respectively.

#### 5.1.1. AR Dataset

**Figure 4:**
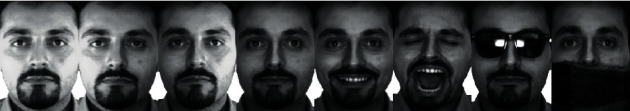
Samples in the AR dataset.

The AR dataset contains more than 4000 positive images, belonging to 126 individuals, with the image size of 120×165. In the experiments, we use a subset of 100 people, 50 men and 50 women, and there are 26 positive images of each person. Among them, 14 images are no blocking images with only changes in expression or light. 6 people wear sunglasses, and 6 people wear scarves. Therefore, the dataset can be divided into two separate parts, and each part contains 13 pictures (7 positive pictures with no blocking and only changes in expression or light, 3 facial pictures with sunglasses, and 3 positive pictures with scarves).  Figure 4 shows some sample images in the AR dataset. We randomly select one part for training and the other for testing. The Gabor wavelet features used in the experiments include 5 scales and 40 features in 8 directions. The deep features used are from the convolution layer in the second part of VGG19, and the number is 128. After PCA dimension reduction, the feature dimensions are 25, 50, 75, 100, and 150.

The experimental results are shown in Table 1. The bold number in each column represents the highest recognition rate under the same condition. Although the recognition rate of M-JSR is not the highest when the dimension is 25, it also remains at the average level. When the dimension is above 50, the recognition rate of M-JSR is higher than that of other methods.

#### 5.1.2. Extended YaleB Dataset

**Figure 5:**
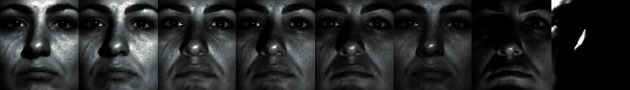
Samples in extended YaleB dataset.

The Extended YaleB dataset consists of 2,414 positive images of size 168×192, in which there are 38 people under different lighting conditions.  Figure 5 shows some sample images from the Extended YaleB dataset. In the experiments, we randomly selected 16 images of each person for training and the rest for testing. The Gabor wavelet features used in the experiment include 5 scales and 40 features in 8 directions. The deep features used are from the convolution layer in the second part of VGG19, and the number is 128. After PCA dimension reduction, the feature dimensions are also 25, 50, 75, 100, and 150.

The experimental results are shown in Table 2. The bold number in each column represents the highest recognition rate under the same condition. The M-JSR method maintains high accuracy rates in all dimensions, only slightly lower than D-AJSR in 50 and 75 dimensions. Compared with the AR dataset, the recognition rates are relatively higher because there is no image with sunglasses and scarf.

### 5.2. Remote Sensing Image Recognition Experiments

**Figure 6:**
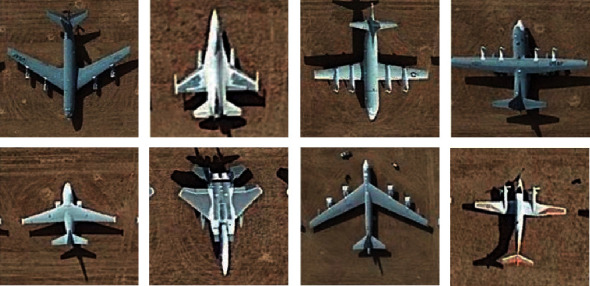
Examples of remote sensing aircraft images.

In this part, we download the remote sensing aircraft images of different shooting times and locations on Google Earth 7.1.8 as the experimental dataset. In the dataset, 375 remote sensing images are classified to 15 aircraft types, as shown in Figure 6. 10 images in each aircraft type are randomly selected for training and 15 for testing. The image size is 170×170. The Gabor wavelet features used in the experiment include 5 scales and 40 features in 8 directions. The deep features used are from the first part of VGG19, and the number is 64. After PCA dimension reduction, the feature dimensions are also 25, 50, 75, and 100. The experiment results are shown in Table 3. The bold number in each column represents the highest recognition rate under the same condition.

It can be seen from Table 3 that M-JSR has better effect than other methods. This is because the addition of Gabor wavelet feature can provide more information in different directions. However, compared with the recognition rates of face images, the recognition rates are relatively lower. It is mainly because many planes leave shadows on the side due to the slanting sun. As a result, the contour of two planes will appear on the feature map when the image feature is extracted, which has great interference to the subsequent recognition.

### 5.3. Comprehensive Analysis of Experiments

In the experiment, when PCA was used in dimensionality reduction, the cumulative variance contribution rates of the 3 datasets were also different, as shown in Table 4. It can be seen that the cumulative variance contribution rates of M-JSR on all datasets is low. The reason is M-JSR uses the mixed features which composed of Gabor wavelet features and deep features, so the energy of feature vectors would not be concentrated during PCA dimensionality reduction. Relatively speaking, the fewer principal components are selected, the lower the cumulative variance contribution rate will be. At the same time, the recognition rates of M-JSR are also low when the feature dimension is low.

In addition to the contribution rates of the cumulative variance, the time efficiency of M-JSR is also calculated on 3 datasets, respectively. The training efficiency results of AR dataset and Extended YaleB dataset are shown in Table 5, and the test efficiency results are shown in Table 6. The unit of time is seconds (s). In these experiments, the images of the AR dataset is more than those of the YaleB dataset, so that the training time and test time required for the AR dataset are more than that of the Extended YaleB dataset.

On the remote sensing dataset, the time efficiency of M-JSR is compared with that of SRC, AJRC, and D-AJSR. The training efficiency results are shown in Table 7, and the test efficiency results are shown in Table 8. The unit of time is seconds (s). As can be seen from Table 7 and Table 8, since M-JSR needs to extract two types of features, it takes more training time and more testing time than the other methods. However, considering the recognition rate, we still think the M-JSR method has its own advantages.

It can be seen from the previous experiments that M-JSR has a good robustness for the illumination change and rotation of the image because of the combination of Gabor wavelet features and deep features. Moreover, when the dataset is small, satisfactory recognition results can also be obtained. In many cases, it is difficult to obtain a large number of target images, and the image quality is generally poor due to the influence of dim light, distortion, and other factors. In this case, M-JSR can also provide accurate identification results.

## 6. Conclusions

For the application requirements of image target recognition, Gabor wavelet features and deep features are introduced into JSR in this paper. The classification framework (M-JSR) has good robustness for deformation, rotation, and light and shade change and can get relatively accurate recognition results with only a few training samples. In M-JSR, two kinds of features are composed into mixed features, in which the weights can be adjusted adaptively. The joint sparse model divides the feature dictionary into public part and private part, which reduces the required storage space and improves the recognition accuracy of the image target. However, because M-JSR needs to extract two characteristics, it takes more time than other methods. Therefore, in the future research, how to take into account the feature expressiveness and extraction speed is a problem that needs to be paid attention. Using lightweight networks [23] for feature extraction is an effective approach.

## Figures and Tables

**Table 1:** Recognition rates (%) on the AR dataset.

Dimensions	25	50	75	100	150
SRC [17]	64.29	81.29	88.43	89.29	90.29
ESRC [18]	63.14	80.43	85.43	86.14	87.29
LR [19]	68.57	84.14	86.00	88.71	88.00
DLRR [20]	75.71	88.14	89.43	91.00	91.86
SDD [21]	75.86	87.29	89.71	91.71	93.00
D-AJSR [22]	67.10	86.00	90.70	94.10	95.10
M-JSR	71.00	88.20	94.60	96.00	96.80

**Table 2:** Recognition rates (%) on extended YaleB dataset.

Dimensions	25	50	75	100	150
SRC [17]	72.98	85.22	88.43	90.48	92.30
ESRC [18]	73.86	85.33	88.37	90.20	91.20
LR [19]	75.97	84.39	88.21	89.09	91.14
DLRR [20]	85.44	89.81	89.92	92.25	93.05
SDD [21]	89.70	92.03	92.41	92.69	92.75
D-AJSR [22]	93.16	96.05	96.84	96.58	97.37
M-JSR	93.42	95.00	96.E68	97.36	97.63

**Table 3:** Recognition rate (%) of remote sensing aircraft images.

Dimensions	25	50	75	100
SRC [17]	62.00	63.56	65.33	66.00
AJRC [14]	70.62	72.00	76.67	78.67
D-AJSR [22]	71.33	75.53	77.33	80.65
M-JSR	74.25	78.67	82.00	82.67

**Table 4:** Cumulative variance contribution rates (%) on different datasets.

Dimensions	25	50	75	100	150
AR [15]	45.32	55.16	57.42	61.20	69.04
Extended YaleB [16]	42.90	59.58	67.91	73.84	82.37
Remote sensing data set	43.42	61.80	75.03	85.45	—

**Table 5:** Training efficiency (s)of different datasets.

Dimensions	25	50	75	100	150
AR [15]	609.150	689.515	813.090	1077.03	1420.16
Extended YaleB [16]	326.109	366.662	409.921	519.172	645.442

**Table 6:** Test efficiency (s) of different datasets.

Dimensions	25	50	75	100	150
AR [15]	1105.84	1273.50	1497.33	1817.91	2899.68
Extended YaleB [16]	642.385	674.836	694.840	749.198	850.541

**Table 7:** Training efficiency (s) of different methods on remote sensing dataset.

Dimensions	25	50	75	100
SRC [17]	1.2649	1.2901	1.2758	1.2833
AJRC [14]	49.734	58.775	78.598	115.08
D-AJSR [22]	63.104	72.078	94.864	128.94
M-JSR	74.053	82.471	101.49	136.11

**Table 8:** Test efficiency (s)of different methods on remote sensing dataset.

Dimensions	25	50	75	100
SRC [17]	4.1456	7.4306	8.1706	9.4669
AJRC [14]	105.14	108.93	113.11	117.32
D-AJSR [22]	121.00	131.29	132.51	134.70
M-JSR	135.62	138.54	142.97	146.77

## Data Availability

All datasets in this article are public datasets and can be found on public websites.
